# Genome-wide study of an elite rice pedigree reveals a complex history of genetic architecture for breeding improvement

**DOI:** 10.1038/srep45685

**Published:** 2017-04-04

**Authors:** Shaoxia Chen, Zechuan Lin, Degui Zhou, Chongrong Wang, Hong Li, Renbo Yu, Hanchao Deng, Xiaoyan Tang, Shaochuan Zhou, Xing Wang Deng, Hang He

**Affiliations:** 1 School of Life Sciences, State Key Laboratory of Protein and Plant Gene Research, Peking-Tsinghua Center for Life Sciences, and School of Advanced Agriculture Sciences, Peking University, Beijing 100871, China; 2 Peking University-Tsinghua University-National Institute of Biological Sciences Joint Graduate Program (PTN), Peking University, China; 3 Guangdong Provincial Key Laboratory of New Technology in Rice Breeding, Rice Research Institute, Guangdong Academy of Agricultural Sciences, Guangzhou 510640, China; 4 Shenzhen Institute of Molecular Crop Design, Shenzhen 518107, China; 5 Guangdong Key Lab of Biotechnology for Plant Development, College of Life Sciences, South China Normal University, Guangzhou 510631, China

## Abstract

Improving breeding has been widely utilized in crop breeding and contributed to yield and quality improvement, yet few researches have been done to analyze genetic architecture underlying breeding improvement comprehensively. Here, we collected genotype and phenotype data of 99 cultivars from the complete pedigree including Huanghuazhan, an elite, high-quality, conventional *indica* rice that has been grown over 4.5 million hectares in southern China and from which more than 20 excellent cultivars have been derived. We identified 1,313 selective sweeps (SSWs) revealing four stage-specific selection patterns corresponding to improvement preference during 65 years, and 1113 conserved Huanghuazhan traceable blocks (cHTBs) introduced from different donors and conserved in >3 breeding generations were the core genomic regions for superior performance of Huanghuazhan. Based on 151 quantitative trait loci (QTLs) identified for 13 improved traits in the pedigree, we reproduced their improvement process *in silico*, highlighting improving breeding works well for traits controlled by major/major + minor effect QTLs, but was inefficient for traits controlled by QTLs with complex interactions or explaining low levels of phenotypic variation. These results indicate long-term breeding improvement is efficient to construct superior genetic architecture for elite performance, yet molecular breeding with designed genotype of QTLs can facilitate complex traits improvement.

Rice (*Oryza sativa*) is one of the most important crops in the world. Increased rice production played key roles in food safety, especially in developing nations from Asia and Africa. Conventional plant breeding has contributed greatly to yield and quality improvements of modern rice^1^,^2^. Yet the time consuming and labor-intensive selection on the basis of observable phenotype, without knowledge of the genetic basis of the selected traits makes conventional breeding unequal to the increasing need of developing new high-yield varieties of rice with resistance to stressful environments and unpredictable climate changes^3^,^4^. In recent decades, the cost-effective next generation sequencing attracted continuing molecular genetic analysis of traits in animal and plant populations^5^, that contributed to better understanding of quantitative traits genetics. The majority of agronomically important traits are controlled by multiple genes each with major or minor effect. These genes are discovered as quantitative traits loci (QTL). Identification of QTLs is very important for modern breeding. Once a QTL controlling a favorable trait is mapped with closely linked markers, it can be introduced to an elite cultivar by hybridization-selection rotations of the recurrent parent with the donor plant, and the progeny inheriting the desirable QTL is selected by using the closely linked marker in each cross, a process known as marker-assisted selection^6^, which highly reduces time and efforts needed for the phenotype evaluation of progeny in successive rounds of selection.

Selective sweep (SSW) analysis is widely used to identify gene of interest by looking for signatures of selection during domestication and subsequent crop improvement^7^ based on modeling allele frequency spectra, linkage disequilibrium and haplotype structure. This method provides useful information for domestication genes in human and plant populations^8^,^9^,^10^,^11^,^12^. For example, eight genes with various functions were identified subject to selection during domestication in maize^12^. However, this method relies on sufficient genetic changes selection accumulated in the past and is not applicable to all crop species, for example, 371 loci identified in a selection screen in sorghum failed finding evidence for selection^13^. Besides, genome-wide association studies (GWAS)^14^ have also been widely used to detect markers associated with traits of interest from a population that are derived from collections of diverse germplasms. The feasibility of GWAS successfully identified genes with major effects on flowering time and pathogen resistance (*FRI, Rpm1, Rps2* and *Rps5*) in *A. thaliana* using 95 accessions^15^,^16^. Thus, GWAS in *A. thaliana* is robust to generate candidate QTLs, but these association studies can not specify genes which are casual for the traits, and an important challenge in terms of eliminating false positive results lies on population structure control^17^,^18^.

Except for identifying more and more genes controlling agronomically important traits, unveiling improvement process largely by artificial selection at molecular level is also vital for modern breeding. Only a few practical evaluations have been analyzed on artificial selection so far^19^, because direct study of this process required significant time investment and fine control over many parameters^20^. Researchers used digital simulation methods to evaluate the effects of artificial selection in finite populations, the bottleneck of artificial selection, and maintenance of the selected loci based on theoretical model^21^,^22^. However, these studies lack sufficient support from practical data, which also leads to the ignorance of some factors including interactions between QTLs and environment effect. And most of these studies were performed in animals^23^, when artificial selection in crops has not been comprehensively analyzed.

Huanghuazhan is an elite, high-quality, conventional *indica* rice that has been widely cultivated in southern China and from which more than 20 excellent cultivars have been derived. Most significantly, a long-term and clear breeding history of Huanghuazhan pedigree makes it convincing to conclude important regions related to crop improvement and breeding principles to facilitate modern breeding. In a previous study, we successfully dissected the genetic composition of Huanghuazhan rice with incomplete pedigree information and identified key regions conserved during its breeding^24^. In this study, we investigated 99 cultivars of the complete pedigree by SNP array analysis to dissect selection signatures during 65-year breeding, identify regions related to rice trait improvement, and further explore how loci were selected corresponding to traits improvement in an *in silico* model. This comprehensive study of improved breeding revealed shortcomings of improved breeding in some traits such as traits controlled by QTLs with complex interaction, and can be adopted to excavate experience from successful breeding cases in the past, which will benefit modern breeding.

## Results

### The breeding of Huanghuazhan pedigree improved grain appearance quality, and kept high yield performance

**Figure 1:**
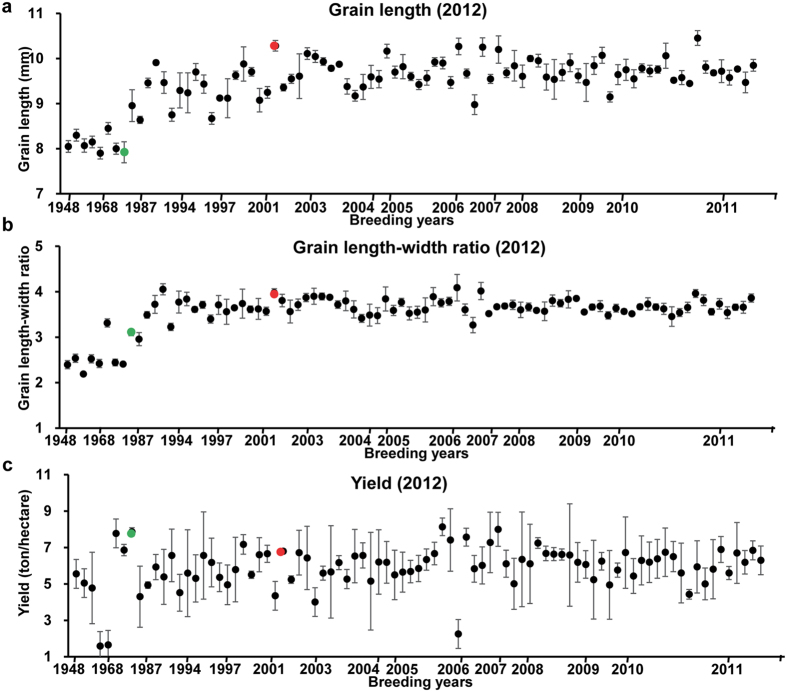
Traits improvement during Huanghuazhan breeding. Black points are cultivars in Huanghuazhan pedigree, the red point is Huanghuazhan, and the green point is Teqing (**a**) Grain length improvement according to breeding year, x-axis is breeding years of the corresponding cultivar, and y-axis is grain length. (**b**) Grain length-width ratio improvement during Huanghuazhan pedigree breeding. (**c**) Grain yield improvement during the breeding.

To confirm traits improvement of Huanghuazhan, we collected 91 cultivars of the complete pedigree and measured 17 traits. Grain quality traits were improved tremendously during its breeding. For example, grain length increased gradually from 8.05 mm (in year 1955) to 8.96 mm (1989), then up to 9.43 mm (2000) and finally kept in an average of 9.67 mm thereafter (Fig. 1a), and grain length-width ratio increased from 2.40 (1955) to 3.10 (1989), then up to 3.71 (2000) and finally kept in an average of 3.68 thereafter (Fig. 1b). Among cultivars in the pedigree, Huanghuazhan was of 10.28 mm in grain length and 3.98 in grain length-width ratio, which was the favorable long grain-length rice consumed in southern china. However, grain yield was changed but not improved at all. As shown in Fig. 1c, grain yield was in an average of 5.92 ± 1.15 tons per hectare in whole pedigree. In relative to Teqing (another elite line in the pedigree) of 7.82 ± 0.22 tons per hectare in grain yield, Huanghuazhan performed 6.18 ± 1.34 tons per hectare in grain yield, which showed small decrease but a still high level (Fig. 1c). These results indicated grain appearance quality of the pedigree were greatly improved during the breeding, while grain yield kept in a high level, which confirmed our former study^24^.

### Four stage-specific selection patterns over 65-year breeding

**Figure 2:**
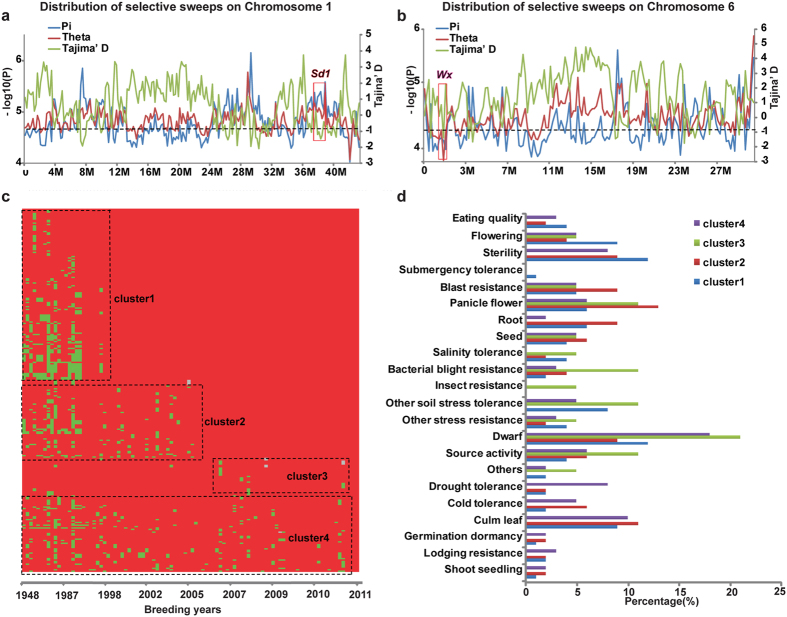
Genetic architecture variation in selective sweeps (SSW). (**a**,**b**) Screening of SSWs on chromosome 1 (**a**) and chromosome 6 (**b**); the y-axis on the left represents the negative logarithm of the Pi or Theta value; the y-axis on the right represents Tajima’s D. Both *Sd1* (**a**) and *Wx* (**a**) were screened out with low Tajima’s D. Pi (blue), Theta (red), and Tajima’s D (green) were calculated in 100-kb windows across one chromosome. (**c**) Full scope of SSW genotype and four variation clusters; each row stands for one SSW block, and each column stands for one cultivar. Cluster 1: selection before 2000; cluster 2: selection before 2007; cluster 3: selection after 2008; cluster 4: selection from 1950 to 2014. Three colors are used to indicate genotypes identical to that of Huanghuazhan (red), different from that of Huanghuazhan (green), or lacking data (gray). (**d**) Major character of reported genes located in SSWs from four different clusters.

To explore signatures of selection during 65-year breeding, we genotyped all 99 cultivars in the Huanghuazhan pedigree with the RiceSNP50 chip^25^. 1,313 SSW blocks were identified by a 5^th^ quantile cut-off of Tajima’s D^26^ as calculated by Variscan^27^ (Dataset S1). The SSW blocks consisted 5.8% of the whole genome (Table S1, Fig. S2). According to the rice genome annotation^28^, 7,723 genes located on the detected SSW blocks. Functional enrichment analysis of these genes showed that they were enriched in the receptor binding, lysosome, and extraordinary stimulus response gene ontologies (Fig. S3a,b). 229 reported genes (Dataset S1) located in these blocks^29^, which included *sd1,* the “Green revolution” gene related to plant height^2^, was detected under high selective pressure in the pedigree (Tajima’s D of −1.356, Fig. 2a), and *wx* locus, that determines the eating quality and amylase-amylopectin ratio of rice^30^, was also under high selective pressure (Tajima’s D of −1.235, Fig. 2b).

Then we clustered the genotypes of the SSW blocks across the 99 cultivars according to the year a cultivar achieved by the breeders, to explore genetic dynamics during the last 65 years. Four distinct patterns of genetic variation were observed (Fig. 2c,d). Genotypes in cluster 1 varied before 1997 and conserved thereafter; genotypes in cluster 2 varied before 2004 and conserved thereafter; genotypes in cluster 3 conserved until 2005 and varied thereafter, and genotypes in cluster 4 varied all through the years from 1948 to 2011. To reveal selection preference underlying these patterns in different breeding periods, we checked reported genes on them^29^ and found that before 1997, genes related to flowering and sterility were preferentially selected, including *OsMSH5*^31^, *rFCA*^32^. From 1997 to 2004, genes related to blast resistance, including *Pib*^33^, were preferentially selected. And after 2004, genes related to other bio-stress were sequentially selected, including bacterial resistance gene *NH1*^34^ and insect resistance gene *OsSUT1*^35^. These results indicated the 65-year breeding consisted several stage-specific selections, including plant type and adaption improvement in the former stage (plant height, flowering time), and recently resistance improvement (biotic and abiotic resistance).

### Conserved Huanghuazhan traceable blocks (cHTBs) are core regions for elite performance of Huanghuazhan pedigree

**Figure 3:**
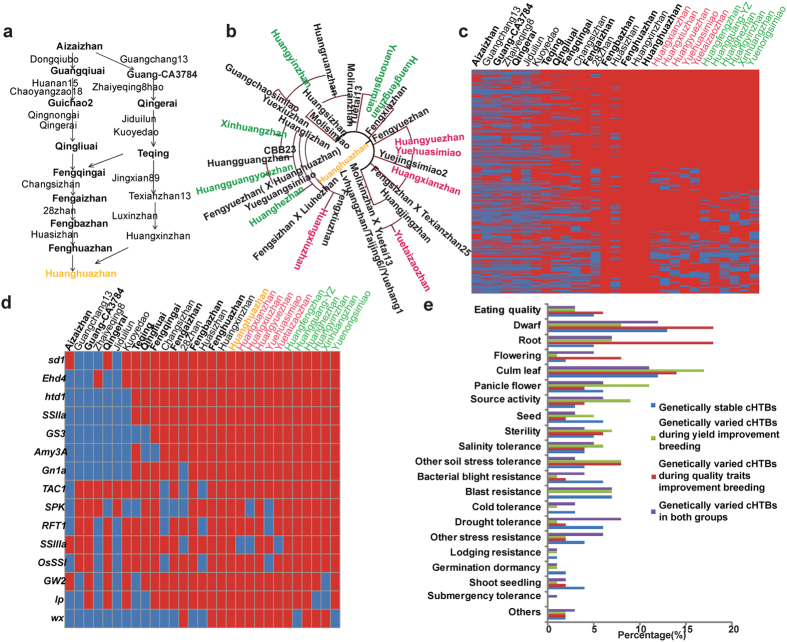
Conservation analysis of Huanghuazhan traceable blocks (HTBs) across the Huanghuazhan pedigree tree and Huanghuazhan derivatives. (**a**) Pedigree tree of Huanghuazhan breeding (The full name of Guang-CA3784 is “Guangchangai3784”, and the full name of Huangguang-YZ is “Huangguangyouzhan”. The abbreviations are used in this figure only). (**b**) Pedigree tree of Huanghuazhan derivative breeding. (**c**) Genotyping of all 1113 cHTBs across the Huanghuazhan pedigree and Huanghuazhan derivatives. (**d**) Genotyping of 13 important agronomical loci. In panels c and d, each row stands for one cHTB, and each column stands for one cultivar, which is ordered by the pedigree tree. Different characters are used to represent upstream cultivars (black), upstream receptors (bold black), Huanghuazhan derivatives superior in quality and plant type (red), and derivatives superior in yield (green). Red blocks represent genotype identical with that of Huanghuazhan, and blue indicates genotype different from that of Huanghuazhan. (**e**) Major character of reported genes located in four groups of cHTBs.

SSW analysis is generally used to identify selection signatures during domestication and subsequent crop improvement^7^, but the conclusion can not explain superiority of Huanghuazhan. To analysis details of Huanghuazhan genetic events and make full use of pedigree relationship, we brought up a genotype tracing method to identify traceable blocks in the pedigree flow. Firstly, we used Haploview^36^ to find 2,840 linkage disequilibrium blocks across all 12 chromosomes (with R^2^ > 0.4), (Table S1), which deemed as haplotype blocks. Then, we compared the genotype of ancestors with that of Huanghuazhan in every haplotype block to determine derivation of Huanghuazhan blocks by searching though the 26 upstream core cultivars (Fig. 3a), and found that 75.6% (2,132 of 2,820) of these haplotype blocks were Huanghuazhan traceable blocks (HTBs), when 23.5% (667 of 2,820) were traced back to Huasizhan in 2000, and 20.3% (576 of 2,820) were traced back to Teqing in 1984, among which 30.7% (177 of 567) were traced back to Aizaizhan in 1955 (Fig. S4). This confirmed our previous study of the Huanghuazhan main pedigree^24^.

Among HTBs identified above, those consistent in more than three sequential breeding generations were deemed as conserved HTBs (cHTBs). Thus, only receptors/donors before Fenghuazhan contributed cHTBs, and 1,113 cHTBs were identified for downstream pedigree study (Dataset S2). After Huanghuazhan, derivative cultivars under subsequent improvement were further divided into two groups according to breeding objectives: a quality group for superior grain quality and plant type, and another group for superior yield (Fig. 3b). We compared the genotype of Huanghuazhan derivative cultivars with that of Huanghuazhan in each cHTB to investigate its consistencies in offspring. Genotypes of 62.1% (692 of 1,113) of cHTBs were highly consistent between Huanghuazhan and cultivars in the quality group, whereas 54% (602 of 1,113) of cHTBs were highly consistent between Huanghuazhan and cultivars in the yield improvement group. The highly consistent regions during grain quality improvement overlapped 81.6% (491 of 602) of those during grain yield improvement (Fig. 3c), suggesting favorable genotypes of these cHTBs (44.1%, 491 of 1,113) are necessary for the superior performance of Huanghuazhan and cannot be replaced during subsequent breeding. Highly-conserved cHTBs in both groups included agronomically important genes such as *sd1*^2^ for plant height, *Ehd4*^37^ for heading date, *htd1*^38^ for high tilling and dwarfism, SS*IIa*^37^ for soluble starch synthesis, *GS3*^39^ for grain size, *Amy3A*^40^ for α-amylase, G*n1a*^41^ for grain number, and *TAC1*^42^ for tiller angle control (Fig. 3d). With regard to 10% (111 of 1113) cHTBs that varied during grain quality improvement breeding, we found genes related to plant type, flowering, and starch synthesis, including *SPK*^43^, *RFT1*^44^, SS*IIIa*^45^, and *OsSSI*^46^ (Fig. 3d). On the other hand, 18% (201 of 1113) of cHTBs that varied during yield improvement breeding included *GW2*^47^ for grain weight and width, *lp*^48^ for panicle size, and *wx*^30^ for eating quality (Fig. 3d). This result corresponded to characteristics of Huanghuazhan traits improvement that the grain quality of Huanghuazhan was greatly improved, whereas its grain yield maintained at a high level, suggesting that variation in these cHTBs is related with corresponding traits improvement. Then, we made function enrichment analysis on annotated genes in these cHTBs (Fig. S11a–d) and checked reported genes^29^ of agronomic importance in the four groups of cHTBs. Genes in genetically stable cHTBs functionally enriched in biological process such as regulation of gene expression, epigenetic, photosynthesis, and cell communications (Fig. S11b), and reported genes functioning in blast resistance and seeds distributed on the cHTBs (Fig. 3e). Genes in genetically variable cHTBs during yield improvement breeding functionally enriched in biological process such as regulation of gene expression, epigenetic, cell cycle, pollination, and growth (Fig. S11c), and reported genes functioning in source activity were included. Whereas genes in genetically variable cHTBs during quality and plant type improvement breeding functionally enriched in chromatin binding, generation of precursor metabolites and energy, respiratory electron transport chain (Fig. S11d), and reported genes functioning in dwarfism and roots were included. And those that varied in both groups related to drought tolerance and other resistance (Fig. 3e).

### Genome-wide association studies identified QTLs in selected regions

**Figure 4:**
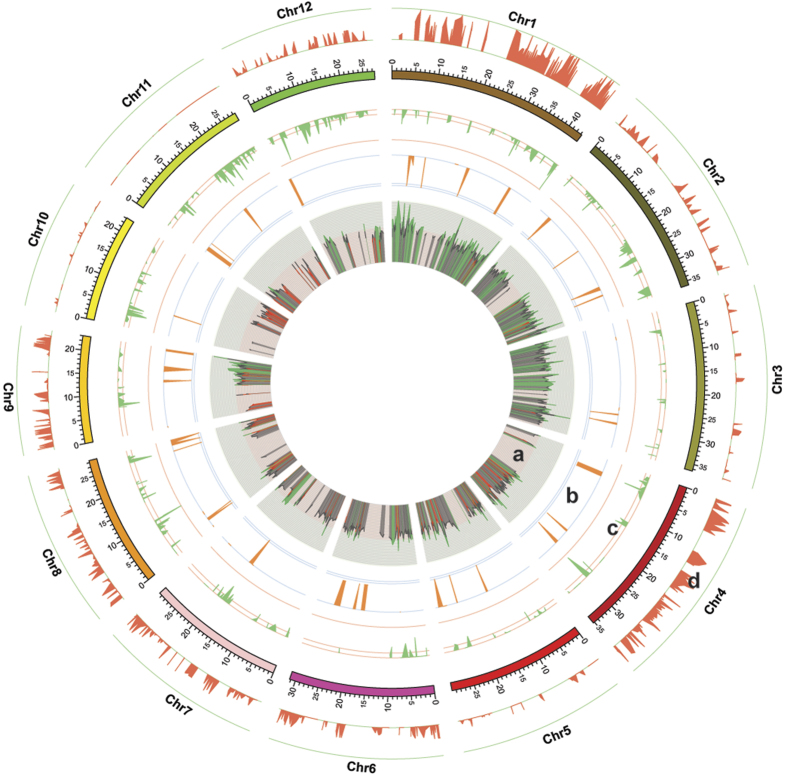
Summary of the genetic architecture of the Huanghuazhan rice (*Oryza sativa*) pedigree. (**a**) The inner circle represents the proportions of functional gene clones per 10 kb on 12 chromosomes (ranging from 0 to 1, where peak height indicates cloned gene abundance). (**b**) The second orange circle depicts proportions of QTL blocks from the association study on data collected in 2012 and 2013 per 1 Mb (ranging from 0 to 1, where peak height indicates cloned gene abundance; the three green lines are the axis, 1^st^ quarter, and 2^nd^ quarter values from the outer layer to the inner layer; the inner most line has a value of 1). (**c**) The third green circle represents the proportions of HTBs per 100 kb over 12 chromosomes (ranging from 0 to 1; the three green lines are the axis, 1^st^ quarter, and 2^nd^ quarter values from the outer layer to the inner layer; the inner most line has a value of 1). (**d**) The fourth red circle represents the proportions of SSWs per 50 kb over 12 chromosomes (ranging from 0 to 1; the four green lines are the axis, 1^st^ quarter, 2^nd^ quarter, and maximum values from the inner layer to the outer layer; the outer most line has a value of 1).

To explain functions of SSWs and HTBs identified above and further identify gene of agronomic importance, we performed genome-wide association studies (GWAS) on 91 cultivars in year of 2012 (Table S2) for 17 important agronomical traits, as well as on 88 cultivars in 2013 for 19 traits (Table S3), and identified 238 QTLs for 17 traits in 2012 and 198 QTLs for 19 traits in 2013 (Dataset S3). Among QTLs identified, 45.4% (108 of 238 in 2012) of the QTLs in 2012 and 44.4% (88 of 198 in 2013) of the QTLs in 2013 overlapped with the SSW blocks in the pedigree (Tables S2 and S3), while 71.4% (170 of 238 in 2012) and 72.7% (144 of 198 in 2013) of these QTLs overlapped with HTBs (Tables S2 and 3, Fig. 4b,c,d). This indicated selected region identified in SSW and HTB analysis related to agronomic traits, and HTB was more powerful than SSW to identify selection signature during pedigree breeding. Besides, many QTLs overlapped with reported functional genes^29^ (69 QTLs in 2012 and 77 QTLs in 2013, Dataset S3). For example, a QTL for plant height in both studies overlapped with *qPH-2*^49^, a gene related to plant height (Fig. S5a,b), which explained 49.7% of height variation in 2012 and 42.2% of that in 2013. A reported QTL *GS3*^39^ for grain size explained 65.0% of grain length variation in 2013 (Fig. S5d), 62.8% of grain length-width ratio variation in 2013 (Fig. S5e), and 65.4% of grain width variation in 2012 (Fig. S5c); another reported QTL *GW2*^47^ for grain weight and width explained 29.1% of of grain width variation in 2012 (Fig. S5c).

QTLs explained 12.0~96.7% of phenotypic variation in 2012 (Table S2) and 23.5~91.9% of phenotypic variation in 2013 (Table S3). Among them, high levels of phenotypic variation explained (PVE) were observed in ten grain width (96.7% in 2012 and 91.9% in 2013) and ten grain length (86.1% in 2012 and 73.7% in 2013). And low levels of PVE were observed in flag-leaf length (49.5% in 2012 and 31.9% in 2013), flag-leaf width (38.6% in 2012 and 33.3% in 2013), flowering time (29.8% in 2012 and 44.6% in 2013), and empty grain number per panicle (44.8% in 2012 and 46.3% in 2013). To determine the relationship between PVE and retained probability of QTLs, we checked genotypic consistencies in Huanghuazhan derivatives. With regards to QTLs explained less than 10% phenotypic variation, genotype of 33.3% (19 of 57 in 2012) and 28.3% (32 of 113 in 2013) QTLs were consistent in Huanghuanzhan derivatives, while for QTLs with PVE between 10% and 30%, genotype of 52.3% (45 of 86 in 2012) and 75.4% (46 of 61 in 2013) QTLs were consistent, and genotype of 68.0% (66 of 97 in 2012), 70.8% (17 of 24 in 2013) QTLs with PVE more than 30% were consistent in Huanghuazhan derivatives (Fig. S6), suggesting QTLs with higher level of PVE were more probably selected and retained in breeding process.

### Distinct selection signatures of QTLs corresponding to four groups of traits improvement

**Figure 5:**
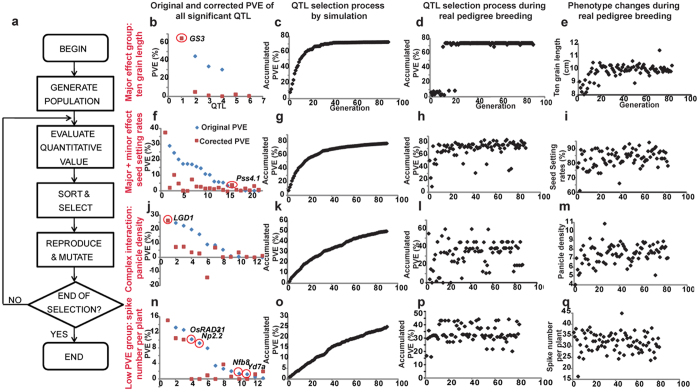
Distinct selection signatures of QTLs corresponding to four groups of traits improvement. (**a**) Scheme of the artificial selection model. (**b**,**f**,**j**,**n**) Original and corrected PVE values of all significant QTLs. (**c**,**g**,**k**,**o**) QTL selection process by simulation; the y-axis represents the accumulated PVE computed from the corrected PVE. (**d**,**h**,**l**,**p**) QTL selection process during real pedigree breeding; the y-axis shows the accumulated PVE based on corrected PVE. (**e**,**i**,**m**,**q**,) Phenotype changes during real pedigree breeding; the y-axis shows the trait value. (**b**–**e**) An example of the major effect group (ten panicle length). (**f**–**i**) An example of the major plus minor effect group (seed setting rates). (**j**–**m**) An example of the complex interaction group (panicle density). (**n**–**q**) An example of the low PVE model (spike number per plant).

Since retained probability of QTLs was related with PVE value of them, we tried to investigate how different QTLs were selected during trait improvement by an *in silico* model. Firstly, we chose 151 QTLs identified for 13 traits in 2013 which showed improving phenotype value to explore to what extent did phenotype improvement correspond to significant changes at individual loci for traits of interest. Firstly, traits were divided into four groups according to distributions of original PVE and corrected PVE values for all QTLs of each trait, including the major effect group (6 traits including ten grain length, ten grain width, thousand grain weight, and etc., were controlled by one major effect QTL) (Fig. S8, Fig. 5b), major plus minor effect group (3 traits including seed setting rates, plant height, and filled grain number per five plants, were controlled by both a major effect QTL and several minor effect QTLs) (Fig. S9, Fig. 5f), the complex interaction group (3 traits including panicle density, filled grain number per panicle and spikelet number per panicle, were controlled by QTLs with complex interactions (especially with negative corrected PVE) (Fig. S10, Fig. 5j), and the low PVE group (one trait including spike number per plant was controlled by minor-effect QTLs) (Fig. 5n).

Then, an *in silico* model was built based on simulation algorithms of artificial selection evaluation^20^,^50^. This model includes four modules (Fig. 4a): (1) generation of the initial population (selecting an initial population of designated size from a hypothetical unlimited population in Hardy-Weinberg equilibrium); (2) quantitative traits of individuals in each generation were calculated from the function of QTL effects and genotype (adjusted with environmental effects); (3) the selection process, in which the less fit members of the population were replaced with the more fit offspring in each generation based on phenotype value; and (4) reproduction of the generations, in which the more fit individual selected in module 3 was used as parents to reproduce offspring considering the effect of inheritability and modified mutation rates. Simulations were randomly repeated 100 times, after which the final simulation output was depicted as the average value from these iterations. PVE was used to assess QTL effects. The breeding process in module 4 was not equivalent to natural breeding when considering artificial selection. Therefore, the modified mutation rate was the product of the natural mutation rate and selection probability. Greater QTL effects were associated with greater differences in corresponding phenotype traits, increasing the selection probability in trait improvement. Thus, a “S”-type curve was set to quantify the relationship between selection probability and PVE, in which the inflection points were 10% and 25% (Fig. S7a). Finally, we assessed effects of population size, heritability, and natural mutation rates on artificial selection. With regard to population size, greater PVE was associated with smaller required population size. When PVE was 40%, a population of 500 was sufficient for breeding, but a population of 5000 was insufficient when PVE was 10% or less. Additionally, population size was indirectly associated with the time required for final saturation breeding; larger populations required shorter breeding periods (Fig. S7b). We concluded that the minimum PVE for a population of 500 was 25%, whereas that of a population of 5000 was 13%. With regard to heritability effects, the selection lines showed decreased saturated PVE relative to 100% heritability. When heritability dropped to 20%, the selection line showed almost no increasing trend (Fig. S7c). Natural mutation rates should be also considered when breeders design experiments. For mutation rates <1e-4, a population of 1000 was insufficient to allow selection for a QTL of 25% PVE within 100 generations (Fig. S7d). These results provide an empirical foundation for experimental design by plant breeders.

**Figure 6:**
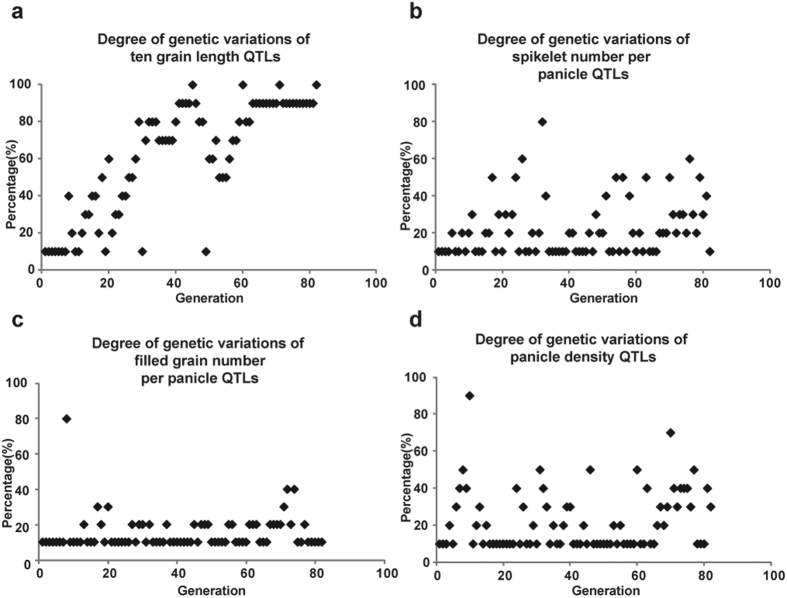
Degree of genetic variations of quantitative traits in different groups. The y-axis represents the percentage of ten recent ancestral cultivars with at most one different QTL compared with the cultivar in each generation. (**a**) An example of the major effect group: ten grain length. (**b**,**c**,**d**) Examples of the complex interaction groups: (**b**) Spikelet number per panicle, (**c**) filled grain number per panicle and (**d**) panicle density.

The model reproduced the selection process of all 13 traits well, revealing distinct selection signatures of traits in the four groups. In the major effect group, the major effect QTL was primarily selected leading to the quick increase of target trait to the maximum (Fig. S8, Fig. 5c,d,e). In the major plus minor effect group, both the accumulated PVE curves and phenotype values showed quick increase primarily and minor increase thereafter for a long time (Fig. S9, Fig. 5g,h,i). In the complex interaction group, genotype values showed a dispersed signature (Fig. 5l), and phenotype values did not increase continuously as QTLs were improved. Theoretically, improvement of the phenotype required simultaneous improvement of multiple loci. The multi-loci selection required the squared population size needed in common breeding, which is impracticable. To test this hypothesis, we checked the number of QTLs sharing consistent genotype with its most recent ten ancestors in each generation, and 27% of cultivars were found to have more than one different QTL in the major effect group. However, for traits belonging to the complex interaction group, 60% (spikelet number per panicle), 95% (filled grain number per panicle), and 80% (panicle density) of cultivars had more than one different QTL from its most recent ten ancestors. As shown in Fig. 6, it is ineffective to gain favorable and stable QTLs by phenotype improvement for traits in complex interaction group, but it is effective for traits in major effect group (Fig. 6a–d). Additionally, there was only one negative interaction combination for the QTLs of spikelet number per panicle, while there were more than two negative interactions for QTLs of filled grain number per panicle, and QTLs of panicle density, suggesting that complexity of negative interactions increased difficulty of QTL improvement during breeding. Traits in the low PVE group were vulnerability to non-genetic factors such as environmental factors, and the phenotypic values were randomly distributed but did not agree with the accumulated PVE curves during artificial selection (Fig. 5p,q). Thus, selection by unaided visual inspection was impossible, and molecular breeding with effective markers could offer an alternative solution to this limitation. In general, long-term breeding improvement is efficient to construct superior genetic architecture of the majority traits, yet molecular breeding should be utilized for specific traits improvement.

## Discussion

A pedigree tree is a full record of sequential cultivars in elite lines with improved traits (receptors) produced by successive crossing of the recurring parent to the donor cultivars. Genotype and phenotype of the long-term pedigree of Huanghuazhan and related breeding materials from year 1948 to 2011, were sufficient and convincing for the SSW, HTB analysis^24^. All methods identified gene of interest, but they had its own emphasis. SSW analysis identifies gene of interest by looking for signatures of selection during domestication and subsequent crop improvement based on modeling allele frequency spectra, linkage disequilibrium and haplotype structure. It is applicable to different kinds of populations, and it served to generally study rice domestication principles in our research. However, HTB method identifies HTBs based on block identity between core cultivar (Huanghuazhan) and the other cultivars in the pedigree with clear relationships, and it served to analyze molecule principle of Huanghuazhan superiority. While GWAS^14^ has been widely used to detect markers associated with traits of interest from a population that are derived from collections of diverse germplasms, and we used it to valid SSWs and HTBs identified above of agronomic importance and further identify QTLs associated with traits of interest. Regions identified from the three methods had overlaps (Fig. 4). Among 7723 genes in SSW blocks, 2843 genes located in HTBs, while there are extra 21265 genes of interest in HTBs (Dataset S2). And 44.4% (88 of 198 in 2013) of QTLs overlapped with SSWs, while 77.8% (154 of 198 in 2013) of QTLs overlapped with HTBs). For example, *GW2*^47^, a QTL for grain weight and width (Fig. 2d), varied during yield improvement breeding, but was not identified by SSW analysis. According to the HTB results, 52% (1113 of 2132) of HTBs were inherited in >3 sequential cultivars (cHTBs), suggesting that long-term crop improvement fixed many loci with agronomic importance. For example, the introduction of a major effect QTL in chromosome 12 increased seed setting rates from 61.0% in year of 1955 to 83.0% in 1992, while further accumulation of 4 minor effect QTLs in chromosome 10 (Chr. 10) in 1997, Chr. 11 in 2001, Chr. 5 in 2005, and Chr. 6 in 2007, increased the value slightly to 89.0%. Furthermore, more than 44% (491 of 1113) of cHTBs are highly conserved in all Huanghuazhan derivatives and are core regions for the superior Huanghuazhan phenotype. For example, the superior genotype in *GS3*^39^ for grain size which conserved in all derivatives, can be utilized in molecular breeding for grain quality improvement. Thus, cHTBs can be utilized as molecular markers in future molecular breeding to maintain the most important genetic architecture of Huanghuazhan.

The *in silico* model was adopted from simulation models for artificial selection evaluation^20^,^51^,^52^, which primarily followed three criteria: (1) reproduction was simulated with a non-breeding or breeding algorithm assuming two alleles and was subject to random point mutations; (2) selection was operated by replacing the less fit members of the population with the more fit members of offspring in each generation; (3) the size of the population and the fraction replaced were constant for each simulation run. These models were able to mimic a variety of the properties of natural selection^20^, but they have several shortcomings, including ignorance of varying effects of loci on traits by assuming additive effects, as well as pure digital simulation without empirical data. Thus we evaluated distributions of original PVE and corrected PVE values for all QTLs of a trait to fully consider interactions among QTLs, and designed the evolving system based on an empirical pedigree breeding program to ensure our simulation was verifiable in every breeding generation. Phenotype and genotype data from the sequential cultivars imprinted by the 65 years of breeding reflected real effects of QTLs on traits, as well as interactions between QTLs, revealing complex interactions among QTLs and low PVE QTLs that cannot be improved by unaided visual inspection.

Distinct selection signatures of QTLs corresponding to four groups of quantity traits improvement concluding from reproductions of 13 improved traits by an *in silico* model suggested breeders should adapt different breeding strategies to promote breeding efficiency. With regard to traits in the major effect group, targeting single major effect loci via conventional breeding and molecular breeding were both effective. For traits in the major and minor effect group, targeting known major effect loci and several minor effect loci via molecular breeding would be more effective than conventional breeding, because selection of minor effect loci took much more time in conventional breeding. However, for traits in the complex interaction group or low PVE group, conventional breeding based on artificial selection was not feasible, but molecular breeding with effective markers eliminating complex interaction QTLs can achieve breeding goals. Thus, this conclusion revealed shortcomings of improved breeding that it was not applicable to improve performance of all traits. Therefore, this model along with distinct selection signatures of QTLs from four groups of traits improvement, will facilitate conventional and molecular breeding. For conventional breeding, this model can be used for planning. The effects of breeding can be estimated by considering traits, population size, generation number, and QTLs. For example, grain width was evaluated in the major effect group; a PVE of 80% for a major effect QTL indicates that grain width will be improved in 20 generations with a population size of 500. However, it will take 80 generations and a population size of 3000 to improve the filled grain number per five plants, which were related to one major effect QTL (18% PVE) and 5 minor effect QTLs. For molecular breeding, the theoretical model can excavate experience from successful breeding cases, which will assist breeders in selection of efficient molecular markers, design of breeding programs for multiple-trait improvement, selection of breeding parents, and other tasks.

## Methods

### Plant materials and phenotyping of the complete pedigree of Huanghuazhan

The pedigree of Huanghuazhan completely records sequential cultivars of elite lines (receptors) produced by successive crossing of the recurring parent to the donor cultivar from 1955 to 2014 (the year when the cultivar was approved by the local rice cultivar assessment committee, and the years mentioned in the paragraph are all “approved year”) (Dataset S4). The complete pedigree includes 2 stages, the Huanghuazhan breeding stage and Huanghuazhan derivative breeding stage. As Fig. 2a shows, the former stage includes the trunk and the branch. The trunk includes 9 receptors and 8 donors, and the branch includes 7 receptors and 6 donors. Aizaizhan (1955) and Huanghuazhan (2005) are shared by the trunk and branch. Teqing (1988) was used as a donor cultivar to improve Qingliuai (1990) in the trunk. Then Huangxinzhan (2002) was used as a donor cultivar to improve Fenghuazhan (2002), producing Huanghuazhan (2005) in the trunk. From Huanghuazhan, more than 20 cultivars have been derived. As Fig. 2b shows, cultivars resulting from derivative breeding include 6 cultivars superior in yield, including Huanghezhan (2009) and Huangyinzhan (2013), as well as 5 cultivars superior in quality and plant type, including Huangyuezhan (2008) and Yuetaizaozhan (2012). Beside, there are 6 cultivars derived from Wushansimiao (2009), 3 cultivars Meixianzhan2 (2006). and 40 cultivars used as breeding materials and medium breeding materials in pedigree breeding of Huanghuazhan, Wushansimiao and Meixianzhan2. There were overlaps cultivars among the pedigree of Huanghuazhan, that of Wushansimiao and Meixiangzhan2, and derivative cultivars of Wushansimiao and Meixiangzhan2 were introduced into Huanghuazhan derivative breeding laterally. Above all, a total of 99 core cultivars and breeding materials were included in the pedigree analysis. Registered cultivars can be referred in china national rice database (http://www.ricedata.cn/variety/). Among the 99 cultivars, we collected 91 cultivars (8 cultivars including Luxinzhan21, Dongqiubo, Guangchangai3784, Guangqiuai, Jiduilun, Kuoyedao, Qingerai, and Qingnongai were not planted) and measured 17 agronomic traits in a randomize block trials design with 3 replications at Zhongluotan locations in Guangzhou, Guangdong (23°23′ 34.27″N, 113°25′ 44.32″E) in 2012, and collected 88 cultivars (11 cultivars including Texianzhan25, Zhenshan97A, Zhenshan97B, Huanan15, Dongqiubo, Guangchangai3784, Guangqiuai, Jiduilun, Kuoyedao, Qingerai, and Qingnongai, were not planted) and measured 19 agronomic traits in a randomize block trials design with three replications at Zhongluotan locations in Guangzhou, Guangdong (23°23′ 34.27″N, 113°25′ 44.32″E) in 2013. The most significant achievement of Huanghuazhan pedigree was the improvement of grain quality. Therefore, we selected grain quality traits primarily, and grain appearance quality traits such as “grain length”, “grain length-width ratio” and “grain width” were included. Except for grain quality related traits, three determinants of grain yield, including thousand-grain weight, filled grain number and effective spike per plant, were included. And plant-type related traits, such as plant height, flag-leaf length and flag-leaf width were also in the traits list.

### Genotyping by RiceSNP50

Ten-day old seedlings of all lines were collected and their genomic DNA was extracted using plant DNA extraction kits (Qiagen, Hilden, Germany). At least 1 μg genomic DNA was used for each cultivar according to the manufacturer’s instructions (Illumina, San Diego). Then all 99 cultivars were genotyped by Rice50K chip^25^ on Illumina HiScan system, with all experimental procedure following the standard protocol provided by Illumina. All raw genotyping data were adjusted by golden standard genotypes designed in our chip platform with GenomeStudio software (Version 2.0, http://www.illumina.com/techniques/microarrays/array-data-analysis-experimental-design/genomestudio.html). And finally a total of 51,479 SNP markers were obtained for the subsequent analysis. Among them, 22,334 SNPs are high quality chip data with genotyping missing rates <80% and minor allele frequency >5% among all lines.

### Identification of SSWs, HTBs and cHTBs

To identify SSW, Pi, Theta and Tajima’s D^26^ were calculated with sliding window of 10 kb across 12 chromosomes with Variscan^27^. and a 5^th^ quantile cut-off of Tajima’s D^26^ was used to identify selective sweeps which may display highly skewed site frequency. Adjacent SSWs within 5 kb were merged to build a whole selected region by an in-house Perl script. SNP sites belonging to each SSW block were combined to construct the genotype of each block. The heatmap plot of SSW was generated using R package pheatmap (https://cran.r-project.org/web/packages/pheatmap/index.html).

To identify HTB, haplotype blocks were primarily computed with an R^2^ cut-off of 0.4 across 12 chromosomes by Haploview^36^ using high quality chip data (Fig. S1; missing rates <80%; minor allele frequency >5%). Then we employed the block strategy with 85% identity threshold of blocks damaged, that is, once the identity of genotype of the researched block between the upstream cultivar and Huanghuanzhan is > = 85%, it was deemed as conserved blocks, which was deemed as HTB in our study. False positives resulted in the strategy were adjusted by integration of the pedigree flow, in which the real HTBs were identified by checking if they were sequentially inherited by the receptors in the order of pedigree flow. And we compared the sequence in each upstream cultivar with the Huanghuazhan genotype in every haplotype block according to the pedigree flow, from Fenghuazhan (2002) to Aizaizhan (1955) (Fig. 2a), to determine the number of HTBs donated by each upstream cultivar.

Among HTBs identified above, those inherited more than three breeding generations were deemed as conserved HTBs (cHTBs). Thus, only receptors/donors before Fenghuazhan contribute cHTBs. And the conservation analysis of these cHTB in Huanghuazhan derivatives was with 85% identity threshold, that is, once the identity of genotype of the researched cHTB between the derivative cultivar and Huanghuanzhan is <85%, it was deemed as cHTB.

### Genome-wide association study

PCA was used to control false positives for the estimation of population structure. Therefore, a compressed mixed linear model (CMLM)^53^ was used to calculate the associations in all analyses using GAPIT software package^54^. The suggestive and significant p thresholds were 1E–04 for each test. It is not a stringent one if compare to previous studies. But it is still a significant one since we have fully considered kinship and population structure in the GWAS model, and our samples size was only 91 or 88. The low sample size reduced statistical power dramatically. Then 10,000 adaptive permutation tests were performed and cut-off was adjusted as listed in Tables S2 and S3. Manhattan plots were generated using GAPIT software package^54^. We have tested the repeatability of the phenotypic data between year 2012 and 2013 (Table S4). Large proportion of the variations was caused by random environmental factors. We did not directly remove the variation between two years across traits, but performed independent GWAS for traits at each year and results of the two years were merged to serve as QTLs for agronomical traits. And the results of 13 traits with improved phenotypic value were used in further improving breeding study.

To estimate the proportion of phenotypic variation explained by QTLs that were detected above, we used a liner model with the *lm* R package. And coefficient of determination (*R*_*s*_^2^) of the model with selected QTL was original PVE for it. Then we used a multi-locus liner model for accumulating selected QTLs with the *lm* R package to adjust PVE of each QTL.

## Additional Information

**How to cite this article:** Chen, S. *et al*. Genome-wide study of an elite rice pedigree reveals a complex history of genetic architecture for breeding improvement. *Sci. Rep.* **7**, 45685; doi: 10.1038/srep45685 (2017).

**Publisher's note:** Springer Nature remains neutral with regard to jurisdictional claims in published maps and institutional affiliations.

## Supplementary Material

Supplementary Information

Supplementary Dataset 1

Supplementary Dataset 2

Supplementary Dataset 3

Supplementary Dataset 4
