# Supplementary material for: Genome-wide study of an elite rice pedigree reveals a complex history of genetic architecture for breeding improvement
Source: Sci Rep. 2017 Apr 4;7:45685. doi: 10.1038/srep45685 (PMC5379486; doi:10.1038/srep45685)
Supplement: Supplementary Information [file srep45685-s1.pdf]

Supporting Information

Article title: Genome-wide study of an elite rice pedigree reveals a complex history of genetic architecture for breeding improvement
Authors: Shaoxia Chen, Zechuan Lin, Degui Zhou, Chongrong Wang, Hong Li, Renbo Yu, Hanchao Deng, Xiaoyan Tang, Shaochuan Zhou, Xing Wang Deng and Hang He

The following Supporting Information is available for this article:

Fig. S1 Distribution of the RiceSNP50 array on 12 chromosomes

Fig. S2 Selective sweep (SSW) distribution on 12 chromosomes

Fig. S3 Gene function enrichment analysis of 7723 genes.

Fig. S4 Proportion of blocks donated by cultivars in the pedigree of the main branch and Teqing branch.

Fig. S5 Manhattan plots for the genome-wide association study

Fig. S6 The relationship between PVE and retained probability in Huanghuazhan pedigree across all QTLs

Fig. S7 Evaluations on effects of population size, heritability, and natural mutation rates on traits improving process

Fig. S8 Quantitative traits in the major effect group

Fig. S9 Quantitative traits in the major plus minor effect group

Fig. S10 Quantitative traits in the complex interaction group

Fig. S11 Functional enrichment analysis of the genes in HTBs and three group of cHTBs.

Table S1 Statistics of block number and coverage of SSWs, haplotype blocks, HTBs and cHTBs over 12 chromosomes

Table S2 Statistics of P-value cut-off and P-value after permutation for GWAS results from 2012, number of QTLs, and numbers of QTLs that overlapped with SSWs and HTBs

Table S3 Statistics of P-value cut-off and P-value after permutation for GWAS results from 2013, number of QTLs, and numbers of QTLs overlapped with SSWs and HTBs

Table S4 Repeatability of the phenotypic data in year 2012 and 2013

Dataset S1 SSWs and agronomic genes in them

Dataset S2 HTBs and agronomic genes in them and gene list with functional annotation in the commonly identified regions and uniquely identified regions

Dataset S3 QTLs identified by GWAS of traits measured in 2012 and 2013 and reported agronomic genes in them

Dataset S4 Approved year and breeding years of 99 cultivars

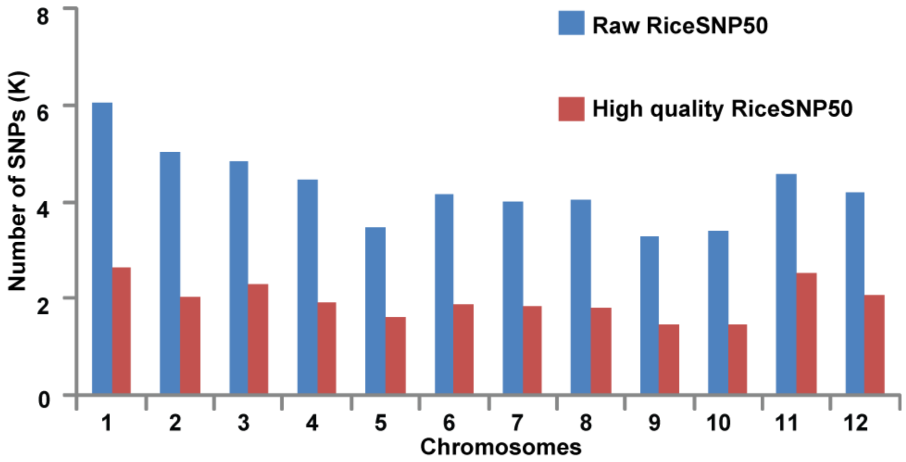

Fig. S1 Distribution of the RiceSNP50 array on 12 chromosomes. High-quality RiceSNP50 represents SNPs with missing rates less than 80% and minor allele frequency (MAF) more than 6% among 99 cultivars.

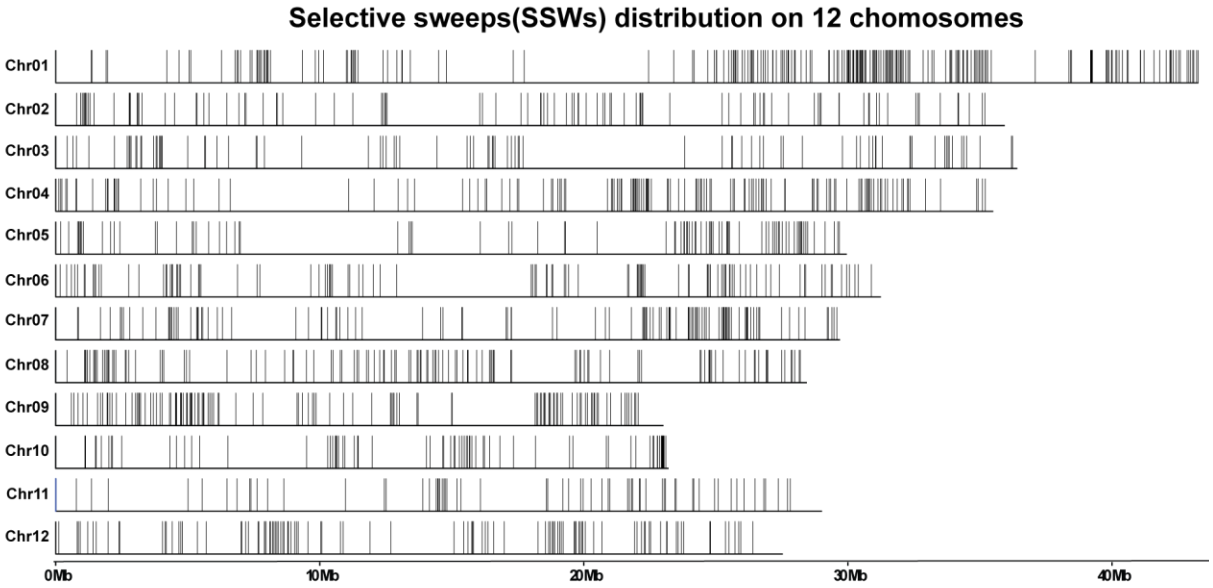

Fig. S2 Selective sweep (SSW) distribution on 12 chromosomes. The black bars show SSWs, and each pass is one chromosome.

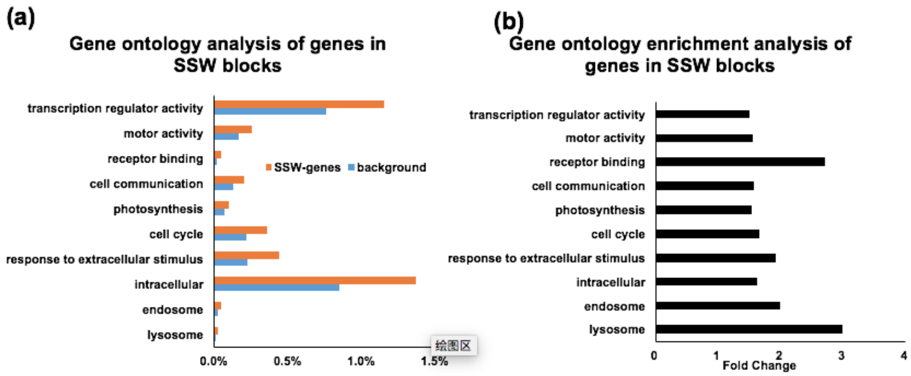

Fig. S3 Gene function enrichment analysis of 7723 genes in SSW blocks.

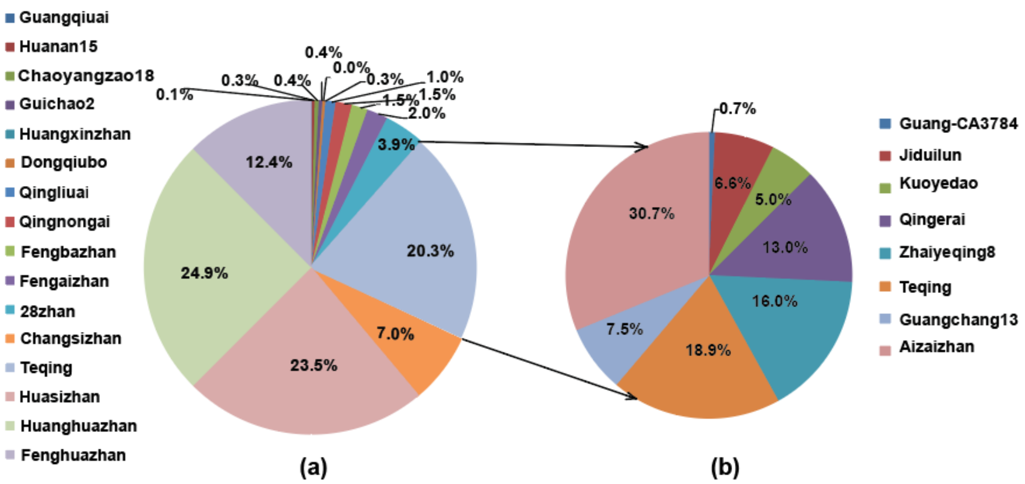

Fig. S4 Proportion of blocks donated by cultivars in the pedigree of the (a) main branch and (b) Teqing branch.

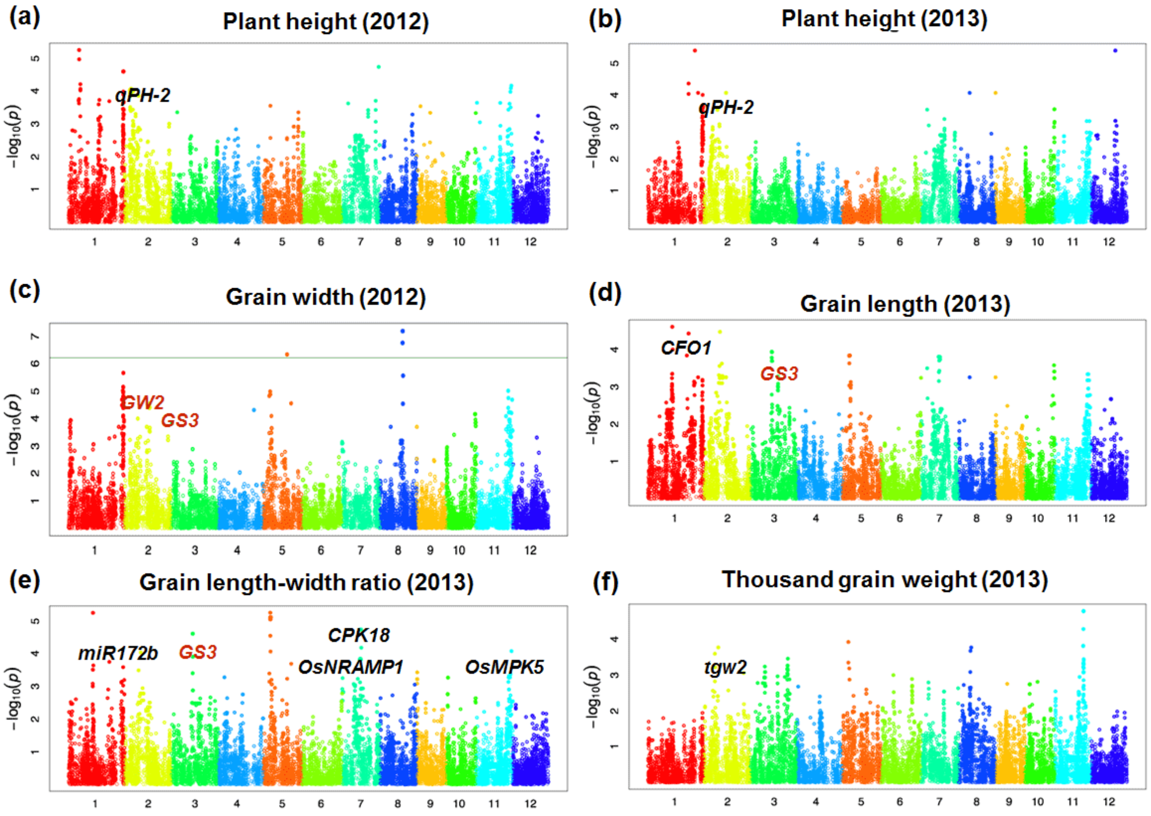

Fig. S5 Manhattan plots for the genome-wide association study. (a) Plant height in 2012; (b) plant height in 2013, *qPH-2* is a major effect QTL for plant height in chromosome 2; (c) filled grain number per five plants; *qPLLN7* located in chromosome 7 functions in pollen fertility; (d) grain length-width ratio, *qLWR-3* is reported to be a QTL for grain length-width ratio in chromosome 3; (e) seed setting rate, *qSS-2* is a QTL for spikelet fertility in chromosome 2; *pss4.1* is a QTL for seed setting rate in chromosome 4; (f) thousand grain weight; *tgw2* is a QTL for thousand grain weight in chromosome 2.

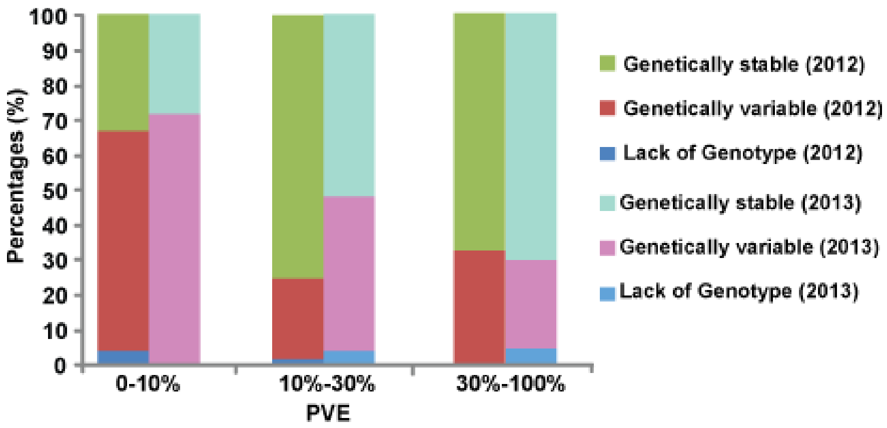

Fig. S6 Evaluations on effects of population size, heritability, and natural mutation rates on artificial selection. The x-axis shows the PVE percentage. The y-axis shows the percentages of QTLs with different genetic events.

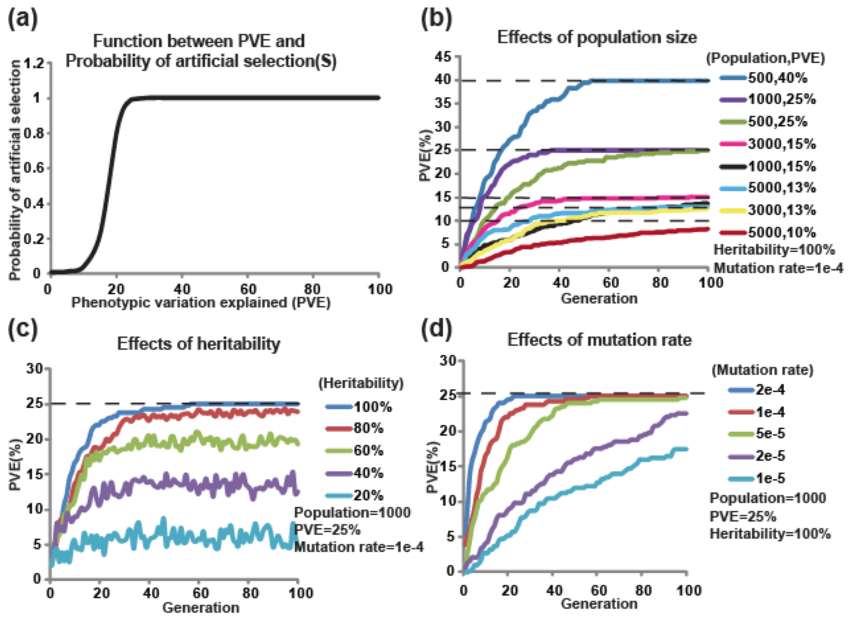

Fig. S7 Evaluations on effects of population size, heritability, and natural mutation rates on traits improving process. (b) Function between PVE and selection probability in artificial selection. (c) Effect of population size on artificial selection. (d) Effects of heritability with fixed population size, PVE, and mutation rates. (e) Effects of mutation rate with fixed population size, PVE, and heritability.

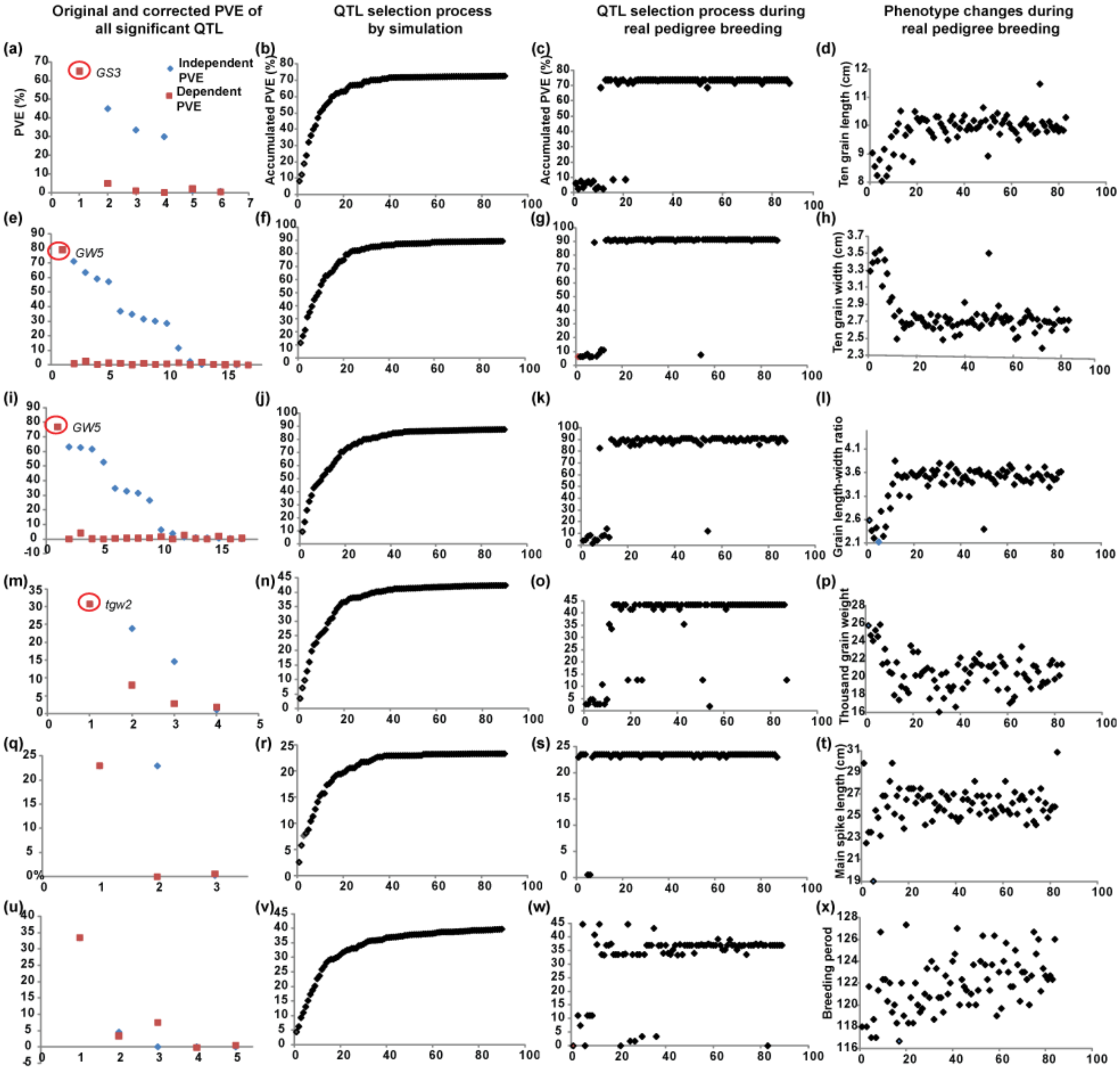

Fig. S8 Quantitative traits in the major effect group. Traits were influenced by one major effect QTL, which was the primary contributor to phenotypic variation, as well as several other QTLs that contributed little to the phenotypic variation. Phenotype traits shown here are most susceptible to artificial selection.

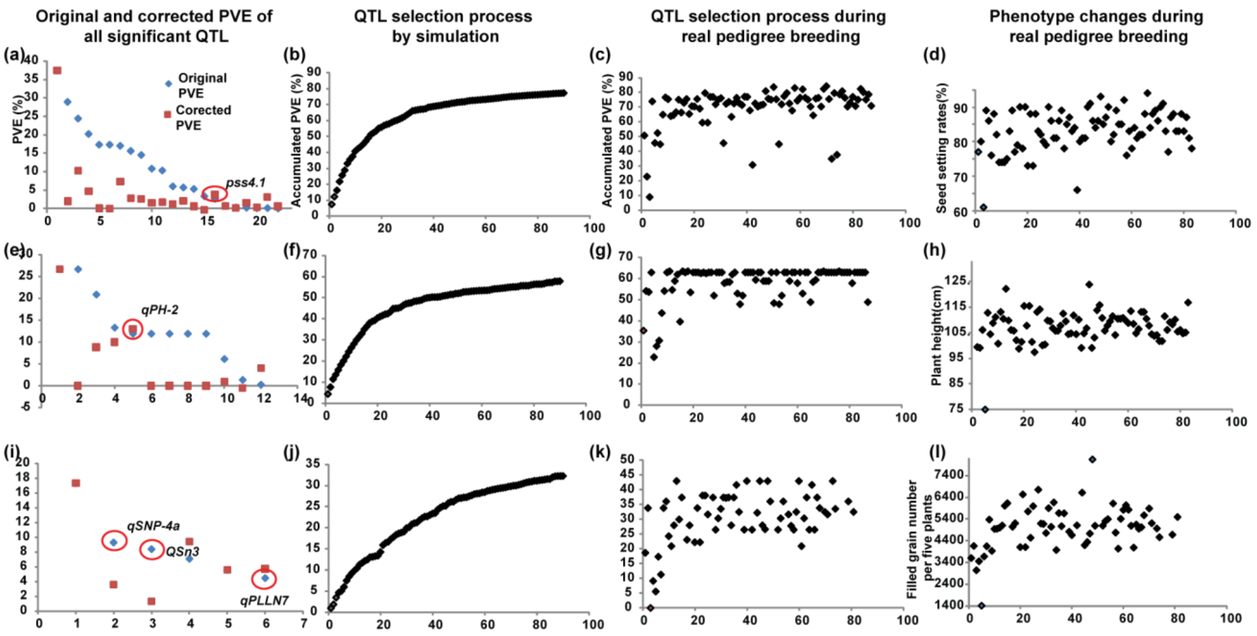

Fig. S9 Quantitative traits in the major plus minor effect group. A major effect QTL and several minor effect QTLs play roles in the phenotypic variation of these traits.

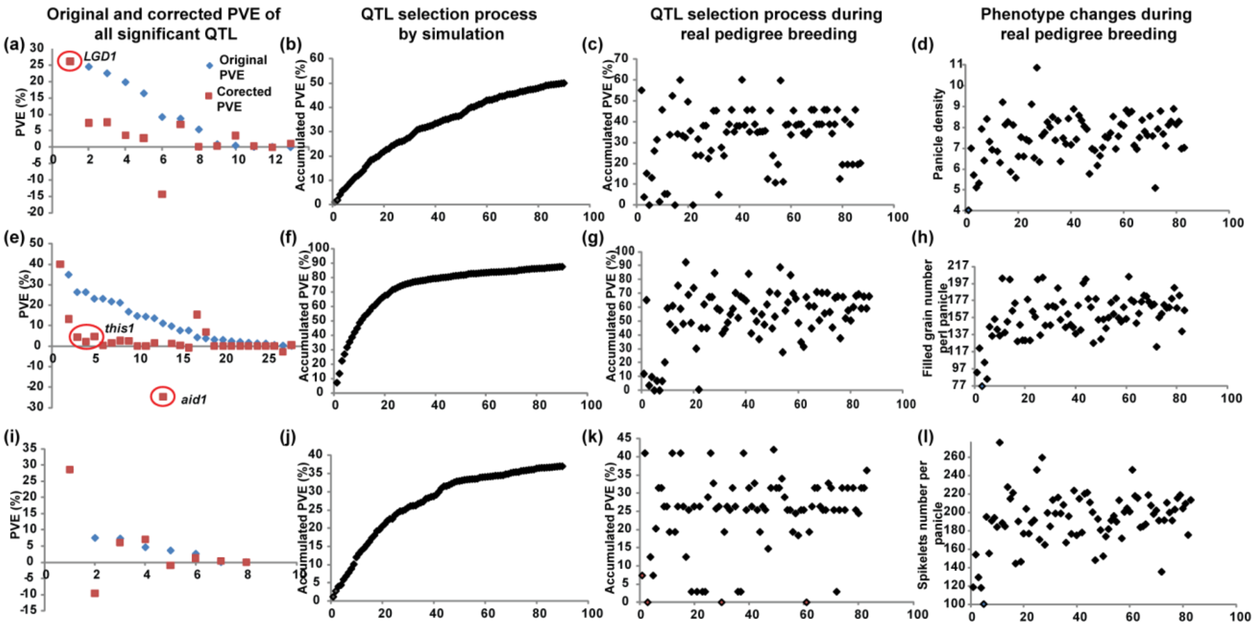

Fig. S10 Quantitative traits in the complex interaction group. There are negative interactions among different combinations of QTLs for these traits.

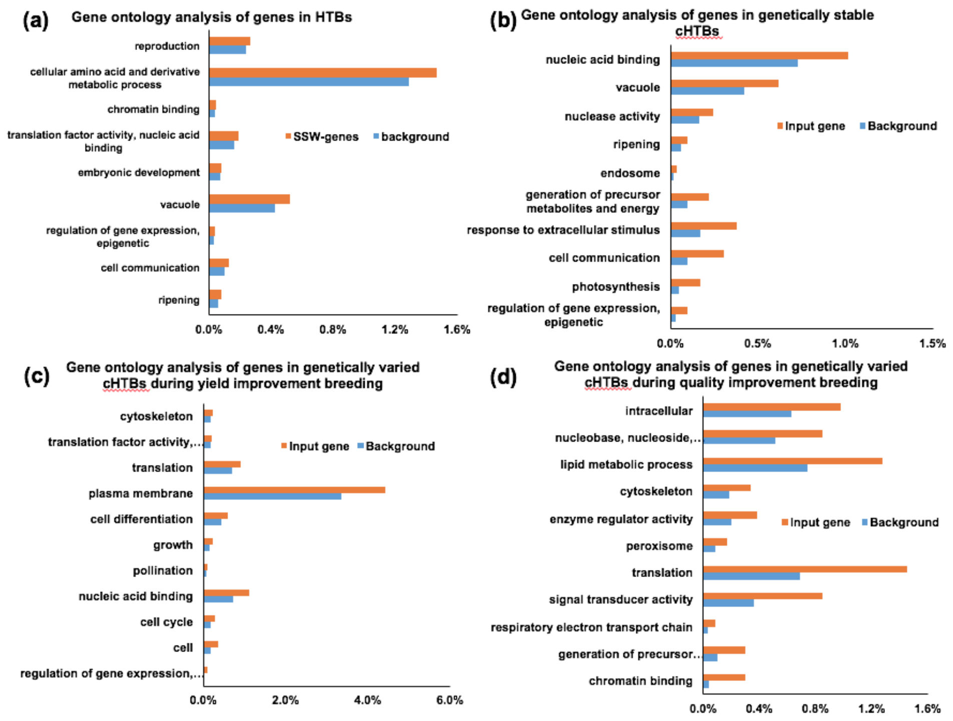

Fig. S11 Functional enrichment analysis of the genes in HTBs and three group of cHTBs.

Table S1 Statistics of block number and coverage of SSW blocks, haplotype blocks, HTBs and cHTBs over 12 chromosomes

**Chromosome**	**SSW blocks number**	**SSW blocks coverage**	**Haplotype-block number**	**HTBs number**	**cHTBs number**	**cHTBs coverage**
Chr1	216	8%	315	238	128	29%
Chr2	99	5%	292	238	112	14%
Chr3	85	4%	218	174	69	16%
Chr4	140	7%	245	123	62	12%
Chr5	85	5%	187	77	40	7%
Chr6	104	6%	174	127	66	18%
Chr7	110	7%	196	160	72	19%
Chr8	118	7%	228	133	86	12%
Chr9	116	9%	189	185	108	26%
Chr10	70	5%	183	175	92	28%
Chr11	65	3%	347	287	152	13%
Chr12	105	6%	266	215	126	20%
Total	1313	6%	2840	2132	1113	18%

Table S2 Statistics of P-value cut-off and P-value after permutation for GWAS results from 2012, number of QTLs, and numbers of QTLs that overlapped with SSWs and HTBs

	**P-value cut-off**	**P-value cut-off after permutation**	**Number of QTLs**	**PVE (%) of all QTLs**	**# SSW-overlap**	**# HTB-overlap**	**# SSW/HTB-overlap**
Flag leaf length	1.0E-04	3.1E-04	5	49.5	2	3	4
Flag leaf width	5.0E-04	4.4E-04	4	38.6	1	3	3
Flowering time	1.0E-04	1.2E-04	2	29.8	1	2	2
Empty grain number	5.0E-03	1.3E-03	3	44.8	1	2	2
Filled grain number	1.0E-04	1.1E-04	2	12.0	0	0	0
Plant height	1.0E-04	1.0E-03	23	86.5	12	12	16
Effective spike per plant	1.0E-04	1.2E-03	19	89.3	10	13	14
Effective spike per acre	1.0E-04	1.9E-03	40	95.2	23	31	36
Yields	5.0E-04	7.3E-04	9	72.6	2	7	7
Grain length-width ratio	1.0E-04	3.0E-03	31	96.9	14	22	24
Seed density	5.0E-03	3.3E-03	9	54.5	1	8	8
Seed setting rates	1.0E-04	2.4E-04	2	35.3	1	1	1
Spike length	1.0E-04	6.3E-04	16	80.9	8	13	13
Ten grain length	1.0E-04	1.5E-03	16	86.1	6	12	12
Ten grain width	1.0E-04	4.0E-03	31	96.7	15	24	25
Thousand grain weight	1.0E-04	4.5E-04	6	56.9	1	4	4
Spike number per five plants	1.0E-04	1.3E-03	20	89.3	10	13	14

Table S3 Statistics of P-value cut-off and P-value after permutation for GWAS results from 2013, number of QTLs, and numbers of QTLs overlapped with SSWs and HTBs

	P-value cut-off	P-value cut-off after permutation	Number of QTLs	PVE (%) of all QTLs	# SSW-overlap	# HTB-overlap	# SSW/HTB-overlap
Flag leaf length	1.0E-03	1.9E-03	4	31.9	2	3	3
Flag leaf width	1.0E-04	1.3E-04	3	33.3	0	2	2
Flowering time	1.0E-03	1.1E-03	5	44.6	1	3	3
Empty grain number per panicle	5.0E-03	3.3E-03	9	46.3	4	8	8
Filled grain number per panicle	1.0E-03	6.8E-03	28	81.3	16	21	23
Empty grain number per 5 plants	5.0E-03	2.4E-03	9	26.7	1	2	3
Filled grain number per 5 plants	5.0E-03	2.0E-03	6	42.9	3	5	5
Plant height	1.0E-04	6.2E-04	12	63.1	4	5	7
Effective spike per plants	5.0E-03	7.9E-03	22	58.9	6	16	16
Grain length-width ratio	1.0E-04	1.1E-03	16	90.9	7	13	13
Seed density	1.0E-03	1.4E-03	13	84.1	4	10	10

	**P-value cut-off**	**P-value cut-off after permutation**	**Number of QTLs**	**PVE (%) of all QTLs**	**# SSW-overlap**	**# HTB-overlap**	**# SSW/HTB-overlap**
Seed setting rate	1.0E-03	5.3E-03	22	84.2	13	19	19
Spike length	1.0E-04	1.9E-04	3	23.5	1	1	2
Spikelet number per panicle	1.0E-03	1.2E-03	8	32.8	3	5	6
Ten grain length	1.0E-04	2.6E-04	6	73.7	4	5	5
Ten grain width	1.0E-04	1.0E-03	17	91.9	10	12	13
Thousand grain weight	1.0E-04	1.9E-04	4	43.4	2	3	4
Grain weight	1.0E-04	2.2E-03	5	40.1	2	1	2
Spike number per 5 plants	1.0E-04	5.0E-03	13	44.2	5	10	10

Table S4 Repeatability of the phenotypic data in year 2012 and 2013

Repeatability of the phenotypic data in year 2012 and 2013 (correlation R^2)	
Flag leaf length	0.41
Flag leaf width	0.28
Flowering time	0.97
Empty grain number	0.33
Filled grain number	0.64
Plant height	0.70
Effective spike per plant	0.11
Spike number per five plants	0.06
Grain length-width ratio	0.85
Seed density	0.60
Seed setting rates	0.53
Spike length	0.64
Ten grain length	0.82
Ten grain width	0.84
Thousand grain weight	0.72
